# Supplementary material for: Comparison and Optimization of DNA Extraction Methods for Human DNA from Dried Blood Spot Samples
Source: Pediatr Rep. 2025 Mar 4;17(2):30. doi: 10.3390/pediatric17020030 (PMC11932244; doi:10.3390/pediatric17020030)
Supplement: Supplementary file 1 [file pediatrrep-17-00030-s001.zip › pediatrrep-3409862-Supplementary Information.pdf]

Supplementary Information (SI)

Contents

SI1: SOP DNA extraction from Dried Blood Spots (using Chelex-100 resin) 2
- Subject 2
- Abbreviations 2
- Basic principles 2
- Reagents 2
- Equipment, disposables and forms 3
- Samples 3
- Method 3
- Literature and support 6

Figure S1: TREC qPCR Comparison of DNA extraction methods 7
Figure S2: TREC qPCR Optimization Roche and Chelex 8
Figure S3: Gel electrophoresis of DNA extracts for DNA integrity assessment 9

SI1: SOP DNA extraction from Dried Blood Spots (using Chelex-100 resin)

Subject

This standard operating procedure (SOP) describes the procedures to extract DNA from Dried Blood Spot samples using a Chelex boiling method.

Abbreviations

DBS	Dried Blood Spot
PBS	Phosphate-buffered saline
SOP	Standard Operating Procedure

Basic principles

This SOP describes a boiling extraction method. First, punched Dried Blood Spots (DBS) are soaked in detergent (Tween® 20) overnight to wash away contaminants like hemoglobin and lysed red blood cells. After an overnight soak, the punches are rinsed in 1X PBS, after which they are brought to boiling temperature (95°C) in a Chelex solution. During boiling, cells on the DBS punch are lysed and DNA is released into the Chelex solution, which chelates metal ions to prevent DNA degradation. After boiling, the extract is centrifuged to spin down the Chelex beads and debris (including the DBS punch). The supernatant contains the DNA and can be aliquoted and used for downstream applications such as qPCR.

Reagents

**Reagent**
Tween® 20
1X PBS
Chelex 50-100 Mesh
100X Tris-EDTA buffer
HPLC

Equipment, disposables and forms

Equipment/disposable/form
Timer
1.7 mL Eppendorf tubes
15 mL Falcon tubes
50 mL Falcon tubes
100 mL glass bottle
Scissors
6-mm DBS puncher
70% ethanol
Paper towel
Heating block
Centrifuge
Vortex
P1000, P200, P20
1250 µL filter tips
200 µL filter tips
Non-filter tips
Pipette boy
Glass serological pipettes
Analytical balance
Weighing boat
Spatula
Rotating magnet
Magnetic stirrer
Tube Rack
96 u-shaped wellplate

Samples

This SOP starts from Dried Blood Spot samples.

Method

In advance

1. Clean the bench using paper towel and 70% ethanol.
2. If no more Chelex solution is available, prepare a 5% (m/v) 50-100 mesh Chelex solution (20 mL):
    a. Make 20 mL 1X Tris-EDTA solution by diluting 200 µL 100X Tris-EDTA stock solution in 19.8 mL HPLC in a 100 mL glass bottle. Invert three times to mix.

b. Weigh 1 g of the Chelex beads using a clean spatula in a weighing boat resting on an analytical balance.
c. Poor the weighed Chelex beads carefully into the 100 mL glass bottle containing 20 mL 1X Tris-EDTA.
d. Mix the solution by making a circular motion to ensure that all of the beads are in the solution
e. Add a rotating magnet into the glass bottle containing the Chelex solution. Put the bottle on the magnetic stirrer and turn it on. The stirrer is used to keep the solution in motion and avoid that the beads will sediment during aliquoting in the next step.
f. Transfer the needed amount of Chelex solution (if n is number of samples: (n x 50 µl) + 500 µl extra) to 1.7ml Eppendorf tubes to preheat it on day 2. To do this, cut a piece of a filter tip (approximately 5 mm) with scissors to ensure the Chelex beads can pass the tip. Before transferring the beads, pipet up and down to ensure the transferred solution contains Chelex beads (visually check).
g. Store the Chelex aliquots and remaining solution at 4°C. (This solution can be stored up until 1 month in the refrigerator).

3. If no more Tween® 20 solution is left, prepare a 0,5% Tween® 20 solution (40 mL, for n=40):

a. Aliquot 44 mL of 1X PBS in a 50 mL Falcon tube using a pipette boy. Perform this step in a flow bench to keep the stock 1X PBS sterile.
b. First, create a 20% working solution by adding 1 mL Tween® 20 stock solution to 4 mL 1X PBS in a 15 mL Falcon tube use a pipette boy. Pipet up and down to mix.
c. Then, create a 0,5% Tween® 20 solution in a 50 mL Falcon tube by adding 1 mL 20% Tween® 20 working solution to 39 mL 1X PBS with a pipette boy. Pipet up and down to mix.
d. Store the 20% and 0.5% solution at room temperature (15-25°C) for up to one month.

Pre-soak (day 1)

1. Punch a 6-mm spot from the DBS sample in a 1.7 mL Eppendorf tube by holding the puncher above the tube so that the punch falls into it. Clean the puncher with 70% ethanol and dry (by punching into paper towel) between samples. Make sure the puncher is dry before punching the next sample.
2. Add 1 ml of 0.5% Tween® 20 solution to the Eppendorf tube containing the 6-mm DBS. Invert three times and incubate at 4°C overnight (e.g. overnight soak from 4 pm day 1– 10 am day 2).

DNA extraction (day 2)

3. After the overnight soak, take the samples from the fridge and remove the 0.5% Tween® soak using a P1000 pipette from the 1.7 mL Eppendorf tubes, leaving the DBS punch in the tube.
4. Aliquot the needed amount (if n is number of samples: (n x 1 mL) + 1 mL extra) of 1X PBS in a Falcon tube using a pipette boy. Perform this step in a flow bench to keep the stock 1X PBS sterile.
5. Add 1 ml of fresh 1X PBS to the punch in the 1.7ml Eppendorf. Invert the tube three times. Then, incubate the tube at 4°C for 30 min.
6. Preheat a heating block at 56°C and at 95°C. Preheat the 5% Chelex aliquots in 1.7 mL Eppendorf tubes prepared on day 1 at 56°C.
7. After the 30 min incubation of the DBS punch, remove the 1X PBS from the Eppendorf tube using a P1000 pipette, leaving the DBS punch in the tube.
8. Add 50µL of the preheated 5% Chelex solution to the DBS punch, using a P200 pipette. To do this, cut the outermost part of a pipette tip (approximately 5 mm) with a pair of scissors so that the opening is wide enough to withdraw the Chelex beads. Pipet the Chelex beads up and down before transferring and visually inspect the pipette tips to make sure that approximately the same amount of Chelex beads are added to each tube.
9. Using a (non-filter) tip, push the DBS punch to the bottom of the 1.7 mL Eppendorf tube so that it is immersed in the Chelex beads.
10. Pulse vortex the sample for 30 s (put the vortex in ‘touch’ mode and put the sample on and off the vortex for 15 times).

11. Incubate the sample at 95°C for 15 min in the pre-heated heating block. During incubation, shortly pulse vortex the sample every 5 minutes (after 5, 10 and 15 minutes) by putting the vortex in 'touch' mode and putting the sample on and off the vortex for 3 times). Put a tube rack on top of the Eppendorf tubes in the heating block to prevent the cap from bursting open. During pulse vortexing, pressure can be released from the tube by slightly lifting the cap (only necessary after the first round of pulse vortexing).
12. After the final pulse vortexing step, centrifuge the sample for 3 min at full speed (13 000 rpm) to pellet the Chelex beads and any degraded paper.
13. Transfer as much of the supernatant containing the eluted gDNA to a new 1.7 ml Eppendorf tube or a 96 u-shaped well plate using a P200 pipette (± 50 µL can be transferred). Avoid transferring the Chelex beads as they can inhibit downstream PCR applications (since the opening of 100/200 µL tips is so small, they usually prevent carry over of Chelex beads).
14. Repeat the steps 12 and 13, but use a P20 pipette in step 13 instead of a P200 pipette to prevent Chelex carry-over. Transfer the additionally recovered supernatant to the same 1.7 ml Eppendorf tube/96-well plate from step 13.
15. Store the DNA extracts at -20°.

Literature and support

1. Neta Simon, Jaclyn Shallat, Corey Williams Wietzikoski, Whitney E Harrington, Optimization of Chelex 100 resin-based extraction of genomic DNA from dried blood spots, Biology Methods and Protocols, Volume 5, Issue 1, 2020, bpaa009, https://doi.org/10.1093/biomethods/bpaa009

Figure S1: TREC qPCR Comparison of DNA extraction methods

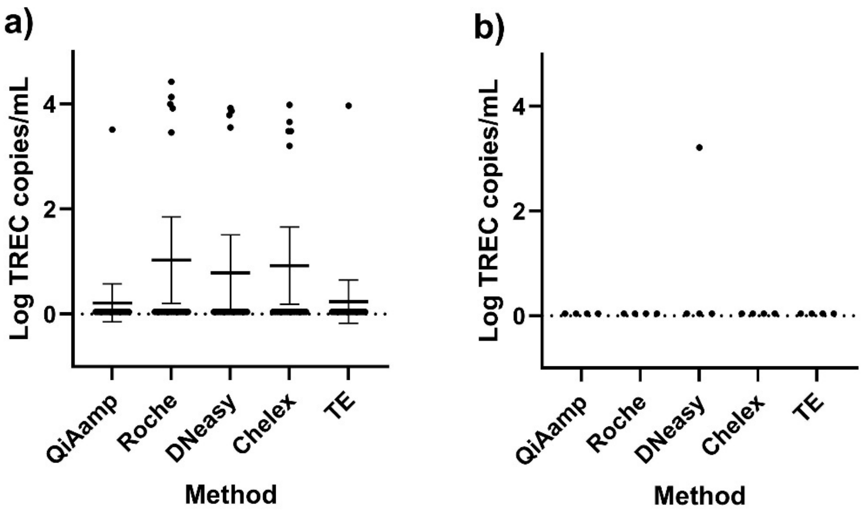

Figure S1: Strip plots of TREC qPCR (log copies/mL) concentrations of the a) DBS samples with mean and 95% CI and b) four blank DBS (negative controls) per DNA extraction method. The means are represented by the middle horizontal lines, and the 95% CI are represented by the error bars. Each dot represents the mean of the duplicate qPCR measurement (log-transformed). No statistical tests were performed due to low number of TREC positive samples. CI = confidence interval. Dashed lines on log(TREC) = 0.

Figure S2: TREC qPCR Optimization Roche and Chelex

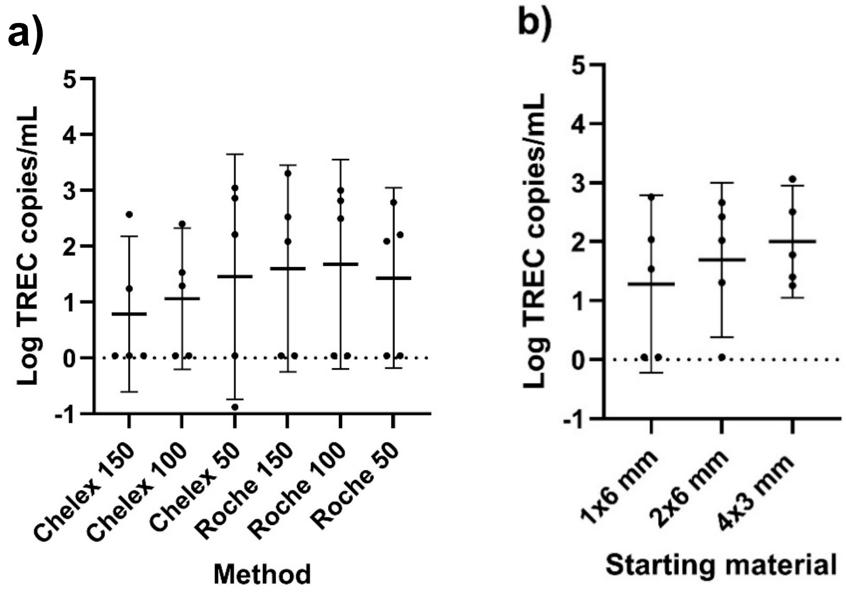

Figure S2: Strip plots with mean and 95% CI of TREC qPCR concentrations (in log copies/mL) for the optimization of Chelex and Roche elution volumes (a) and of Chelex starting material (b). The means are represented by the middle horizontal lines, and the 95% CI are represented by the error bars. Each dot represents the mean of the duplicate qPCR measurement (log-transformed). No statistical tests were performed due to low number of TREC positive samples. Dashed line on log(TREC) = 0. ns = not significant, ** = p-value < 0.01, *** = p-value < 0.001, CI = confidence interval. Dashed line on log(TREC) = 0.

Figure S3: Gel electrophoresis of DNA extracts for DNA integrity assessment

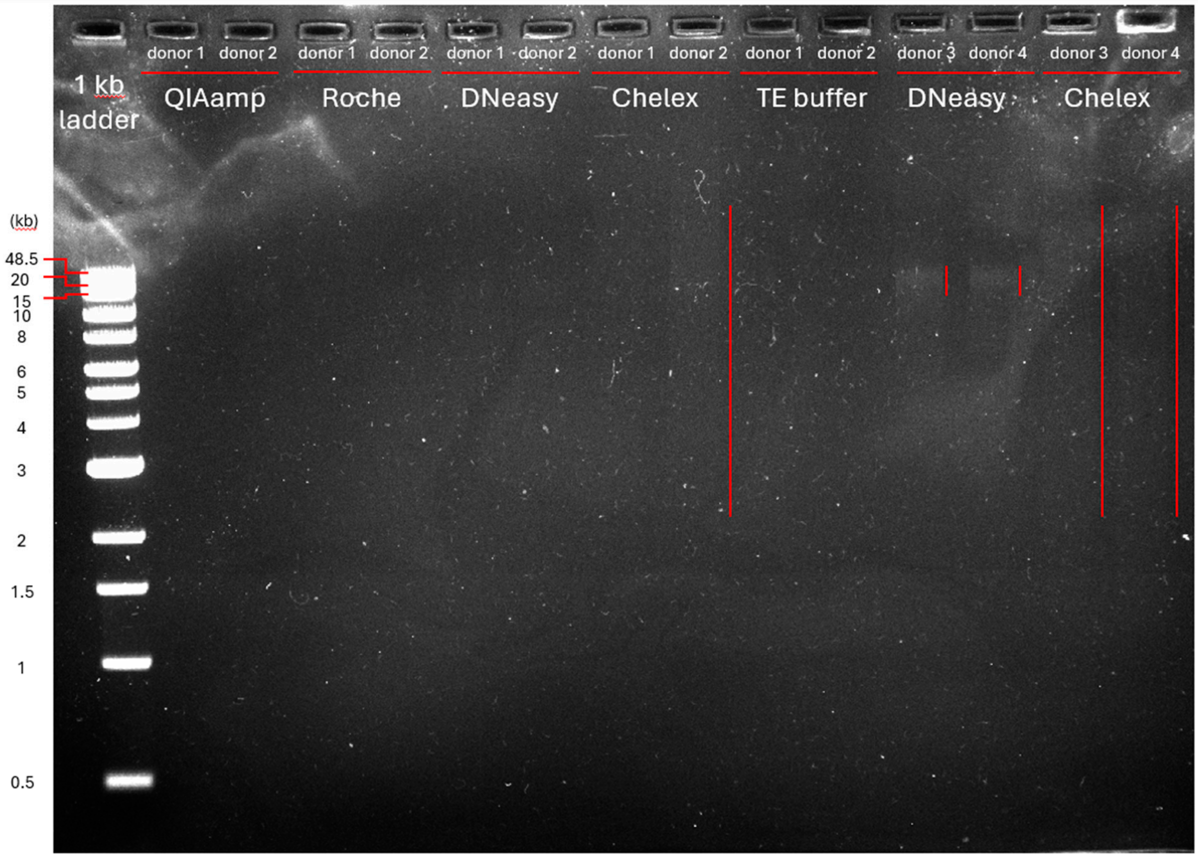

Figure S3: Gel electrophoresis of DNA extracts for integrity assessment. For each of the five DNA extraction methods, extracts from two donors were selected, and an additional two extracts were included for DNeasy and Chelex. Red vertical stripes indicate the signals of smears or lines, faintly visible on the gel. The DNA ladder is visible on the left side of the image. Abbreviations: kb = kilobase.
